# Interventional recanalization therapy in patients with non-cirrhotic, non-malignant portal vein thrombosis: comparison between transjugular versus transhepatic access

**DOI:** 10.1007/s00261-022-03411-w

**Published:** 2022-01-12

**Authors:** Nabeel Mansour, Osman Öcal, Mirjam Gerwing, Michael Köhler, Sinan Deniz, Hauke Heinzow, Christian Steib, Martin K. Angele, Max Seidensticker, Jens Ricke, Moritz Wildgruber

**Affiliations:** 1 Department of Radiology, University Hospital, LMU Munich, Marchioninistr. 15, 81377 Munich, Germany; 2 Institute of Clinical Radiology, University Hospital Muenster, Muenster, Germany; 3 Department of Gastroenterology and Hepatology, University Hospital Münster, Münster, Germany; 4 Department of Medicine I, Hospital of the Merciful Brothers, Trier, Germany; 5 Department of Medicine II, University Hospital, LMU Munich, Munich, Germany; 6 Department for General, Visceral and Transplantation Surgery, University Hopsital, LMU Munich, Munich, Germany

**Keywords:** Portal vein thrombosis, Recanalization, Transjugular intrahepatic portosystemic shunt, Thrombolysis

## Abstract

**Purpose:**

To compare the safety and outcome of transjugular versus percutaneous technique in recanalization of non-cirrhotic, non-malignant portal vein thrombosis.

**Methods:**

We present a retrospective bicentric analysis of 21 patients with non-cirrhotic, non-malignant PVT, who were treated between 2016 and 2021 by interventional recanalization via different access routes (percutaneous [PT] vs. transjugular in transhepatic portosystemic shunt [TIPS] technique). Complication rates with a focus on periprocedural bleeding and patency as well as outcome were compared.

**Results:**

Of the 21 patients treated (median age 48 years, range of 19–78), seven (33%) patients had an underlying prothrombotic condition. While 14 (57%) patients were treated for acute PVT, seven (43%) patients had progressive thrombosis with known chronic PVT. Nine patients underwent initial recanalization via PT access and twelve via TIPS technique. There was no significant difference in complete technical success rate according to initial access route (55.5% in PT group vs. 83.3% in TIPS group, *p* = 0.331). However, creation of an actual TIPS was associated with higher technical success in restoring portal venous flow (86.6% vs. 33.3%, *p* = 0.030). 13 (61.9%) patients received thrombolysis. Nine (42.8%) patients experienced hemorrhagic complications. In a multivariate analysis, thrombolysis (*p* = 0.049) and PT access as the first procedure (*p* = 0.045) were significant risk factors for bleeding.

**Conclusion:**

Invasive recanalization of the portal vein in patients with PVT and absence of cirrhosis and malignancy offers a good therapeutic option with high recanalization and patency rates. Bleeding complications result predominantly from a percutaneous access and high amounts of thrombolytics used; therefore, recanalization via TIPS technique should be favored.

## Introduction

Portal vein thrombosis (PVT) is a rare condition with a reported prevalence of 3.7 per 100.000 population, and half of the cases occur in a patient without liver cirrhosis or malignancy [1]. In contrast to cases with malignancy or cirrhosis, were the outcome is primarily dependent on the underlying disease, non-cirrhotic and non-malignant PVT have a different pathogenesis and the PVT itself is decisive for the prognosis of the patient [2]. Similar to thrombosis in other vessels, non-cirrhotic and non-malignant PVT is related to one or more features of the Virchow’s triad [3–5]. Although PVT without has better survival rates than PVT in patients with cirrhosis or malignancy [1], it is associated with a significant risk of venous congestion of the gut in the acute period and complications related to portal hypertension in the long term, especially life-threatening variceal bleeding, requiring lifelong specialized care [6, 7]. The rarity of this condition precludes large-scale controlled trials, and the treatment algorithm of non-cirrhotic and non-malignant PVT has not been standardized. The American Association For Study of Liver Diseases currently (AASLD) recommends the consideration of interventional portal vein recanalization (PVR) in patients with acute PVT and impending intestinal ischemia [2]. In patients with chronic PVT and recurrent bleeding and/or refractory ascites not manageable medically or endoscopically, interventional PVR followed by TIPS is also currently recommended [2]. Anticoagulation for six months is recommended in patients with reversible etiologies of PVT, life-long in patients with thrombophilia [8].

Several case series have shown that interventional recanalization with thrombolysis via superior mesenteric artery, percutaneous transhepatic portal vein thrombolysis [9] or thrombectomy [10], and recanalization via TIPS access with additional TIPS placement [11, 12] have high technical and clinical success rates, especially in patients with a deteriorating clinical condition and persistent symptoms of portal hypertension despite anticoagulation (impending bowel ischemia or infarction). Recently, a multicenter study has compared the outcome of medical and interventional therapies in non-cirrhotic and non-malignant PVT and showed significantly higher recanalization rates after interventional treatment (37% vs. 71%, *p* < 0.001), despite the higher thrombus burden in the interventional arm at baseline [13]. These results suggest a wider use of interventional procedures in patients with non-cirrhotic and non-malignant PVT. However, further investigation of the optimal approach and technique for interventional recanalization still needs to be clarified, as severe complications including periprocedural bleeding have been reported. The aim of this retrospective, bicentric cohort study was to evaluate the outcome of interventional therapies in patients with non-cirrhotic and non-malignant PVT, and thereby compare the safety and efficacy of different portal vein access routes.

## Materials and methods

### Study design

This observational study includes a retrospective bicentric analysis of the clinical course in a total of 21 patients with non-cirrhotic and non-malignant PVT, who were treated at two tertiary care university hospitals with experience of > 50 TIPS procedures annually from the year 2016 to 2021. Preliminary results of the cohort treated at Center 1 have already been published in a first case series [14]. The study was approved by the institutional review board (Protocol number 2016-046-f-S). Informed consent was waived due to the retrospective character of the study.

### Diagnosis and definitions

PVT was confirmed by contrast-enhanced CT in all patients and was graded according to the previously published grading system [13]: grade 1: incomplete occlusion of the vessel lumen; grade 2: complete occlusion or extended thrombosis; grade 3: the presence of cavernous transformation. PVT was considered acute when symptoms developed less than 60 days before presentation, and there is no radiological or endoscopic evidence of collateral circulation [2, 15–17]. Clinical and laboratory investigations were completed to identify the etiology of PVT, including screening for prothrombotic disorders. All patients were discussed in multidisciplinary rounds, including gastroenterology, interventional radiology, and liver/transplant surgery, and treatment decision was made by consensus in every case.

All patients with grade 2 thrombosis were heparinized with the aim of partial thromboplastin time (PTT) of 60–80 s after the establishment of the diagnosis. Portal vein recanalization procedures, either percutaneous (PT) or via TIPS access, were done under general anesthesia. PT access was preferred for recanalization in the initial procedure, except for the cases with ascites. Transjugular access as the first choice instead was performed in case of technical or clinical failure of PT access or in the presence of ascite due to increased risk of abdominal bleeding.

Technical success was described as complete recanalization of the entire portal venous system or complete bypass of the thrombus via a TIPS. In patients with residual thrombus causing less than a 25% decrease in the lumen, the outcome was described as a partial technical success and the rest of the cases as technical failure.

### Interventional technique and concomitant treatments

Portal vein interventions were performed by interventional radiologists with > 5 years of experience in portal venous interventions. Puncture of the thrombosed portal vein was performed under ultrasound guidance either percutaneously (PT) or via the transjugular route in TIPS technique [11]. In case of inadequate visualization of the portal vein in ultrasound to guide TIPS puncture (*n* = 1), a guidewire was advanced as a fluoroscopic target into the portal vein via percutaneous puncture of the splenic vein. In case of percutaneous access, a 9F sheath was introduced into the portal vein, in case of TIPS access a 10F sheath was inserted. Aspiration thrombectomy was done in all patients with a large-bore aspiration catheter (CAT-8 Indigo Catheter, Penumbra Inc., Alameda, California, USA). In both centers, aspiration thrombectomy was performed as the initial step and proceeded to local thrombolysis in case of incomplete recanalization. Pulse spray thrombolysis was done using dedicated 4F spray lysis catheters with 10–20 cm side holes at a dose of 1 mg/h rt-PA. If overnight thrombolysis was performed, patients were monitored on intensive care units, and control angiography was done on the following day. Balloon angioplasty and additional stenting of the portal vein, superior mesenteric and splenic vein were performed when needed to establish or maintain portal venous flow.

**Fig. 1:**
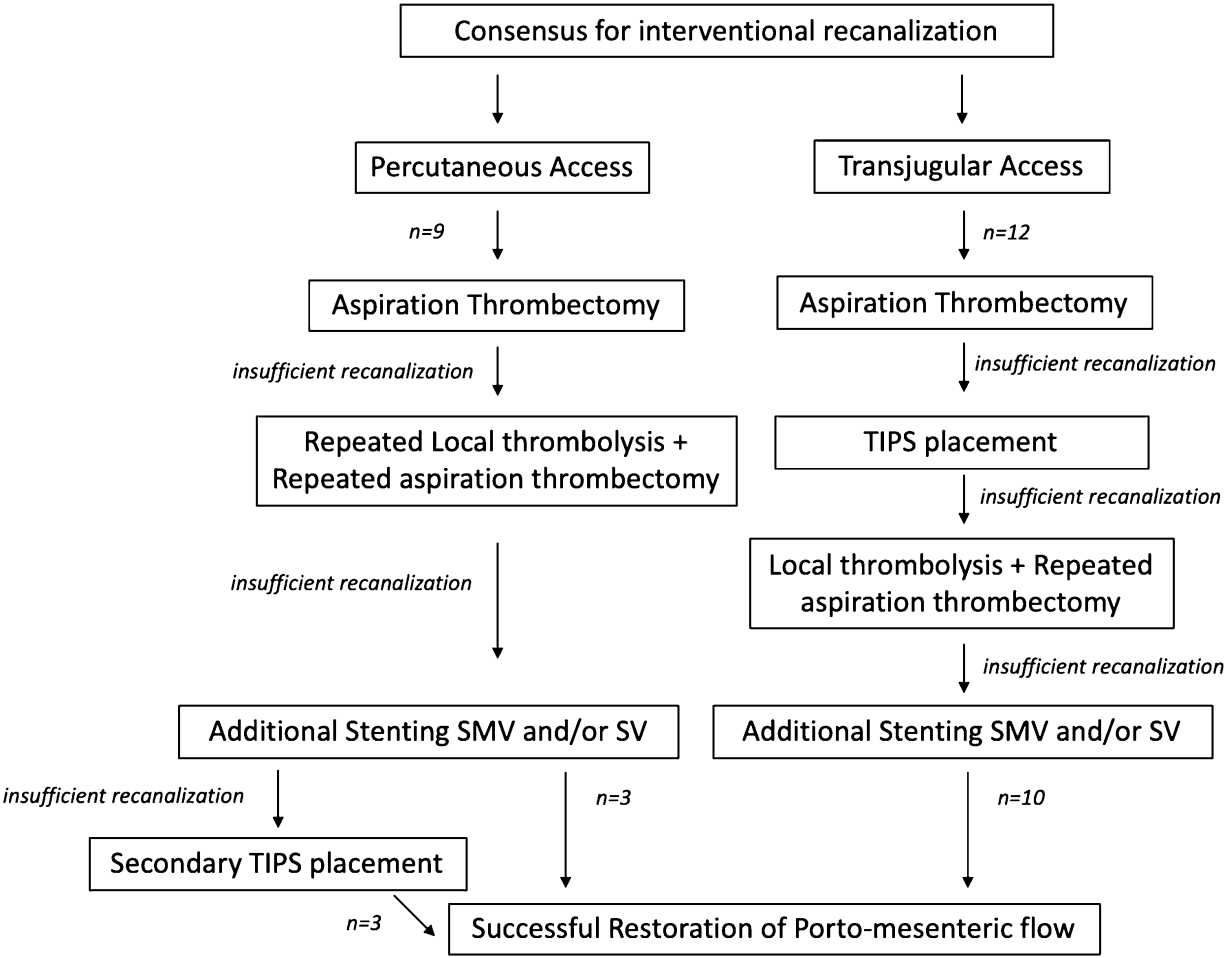
Algorithm of interventional therapy in patients with non-cirrhotic, non-malignant portal vein thrombosis

This step was followed by the creation of TIPS in patients with transjugular access, and in patients with insufficient recanalization after percutaneous thrombus aspiration, local thrombolysis and repeated aspiration were performed. This step was combined with rheolytic thrombolysis using the AngioJet rheolytic thrombectomy system in the power pulse mode (Boston Scientific, Marlborough, MA, USA) in some patients. Thrombolysis was also performed in patients with an inadequate flow after TIPS creation. In patients with insufficient inflow due to thrombosis in SMV or SV, visceral stenting was performed. In case of failed complete recanalization in patients with initial percutaneous access, secondary TIPS was created (Fig. 1).

All patients received therapeutic anticoagulation with unfractionated heparin during treatment (target PTT 60–80 s). Following complete or partial recanalization heparinization was switched to oral anticoagulation with phenprocoumon for at least six months followed by 100 mg/day aspirin monotherapy for lifetime. For patients with an underlying prothrombotic mutation, lifelong oral anticoagulation was initiated. In these cases, novel oral anticoagulants were applied instead of phenprocoumon.

### Statistical analysis

Preprocedural patient characteristics and technical details were grouped as categorical or nominal variables. The total dose of lysis in patients with PT and TIPS access were compared using the non-parametric Mann–Whitney *U* test. Univariate analysis of the relationship between periprocedural characteristics and technical success and bleeding was performed. Factors with a *p*-value < 0.1 were included in multivariate analysis using binary logistic regression. A *p*-value < 0.05 was considered significant. Statistical analysis was performed using R statistical software (R version 3.6.3).

## Results

### Study population

21 patients presenting with non-cirrhotic, non-malignant PVT were treated according to the interventional recanalization scheme described in Fig. 1. Baseline characteristics of the patients are summarized in Tables 1 and 2. The 21 patients included were a median of 48 years, ranging from 19 to 78 years at the time of treatment. An underlying prothrombotic condition was identified in seven patients (33%), of these three had a Janus kinase 2 (JAK2) mutation (14%). In total, 14 patients were treated for acute PVT (57%) with median symptom onset to first intervention of 13 days (range of 3–49). Seven patients had chronic PVT with presence of cavernous transformation of the portal vein and were treated electively due to progressive thrombosis and escalating symptoms related to the increase of portal venous pressure, refractory to medical therapy (grade 3). Clinical presentations consisted of one or a combination of symptoms including abdominal pain (*n* = 12), esophageal varices (*n* = 7) leading to hematemesis (*n* = 3), ascites (*n* = 9), and paralytic ileus (*n* = 1). In the *n* = 7 patients with accompanying ascites treated at Center 1, the ascites was compensated in five patients, allowing for a percutaneous approach. Most of the patients treated in Center 1 underwent portal vein recanalization via percutaneous access (75%). In Center 2, the transjugular recanalization route in TIPS technique was performed in 77% of the cases. Two patients with acute PVT and venous congestion had to undergo open surgery before initiation of interventional treatment, one patient with resection of a gangrenous ileum segment, the other patient underwent emergent splenectomy due to rupture of the splenic capsule [18].

**Table 1:** Patient characteristics and extent of thrombosis

	PT access	TIPS access
Age (median years)	46 (24–78)	51 (19–74)
Sex (male/female)	7/2	8/4
Onset		
Acute (grade 2 PVT)	6	8
Chronic (grade 3 PVT)	3	4
Etiology		
Septic/inflammatory	2	2
JAK2-mutation/MPN	1	2
Other prothrombotic conditions	2	2
Others/unknown	4	6
Clinical manifestation		
Ascites	6	3
Abdominal pain	6	6
Esophageal varices	1	6
Hematemesis	1	2
Paralytic ileus	1	0
Extent of thrombosis		
Intrahepatic + extrahepatic occlusion of PV	6	8
Only intrahepatic	0	0
Only extrahepatic	3	4
Involvement of VMS	8	9
Involvement of SV	4	8

*PT* percutaneous access, *TIPS* transjugular intrahepatic portosystemic shunt, *JAK2* janus kinase 2, *MPN* myeloproliferative neoplasm, *CT* computed tomography, *PV* portal vein, *SMV* superior mesenteric vein, *SV* splenic vein

**Table 2:** Patient history

Patient number	Age (years)/sex	Route of intervention	Onset	Initial symptoms	Etiology and risk factors	Time of symptoms to admission (days)	Time of symptoms to Intervention (days)	Length of hospital stay (days)	Last Follow-up (days)	Patency reached
1	48/F	PT	Acute	Abdominal pain, paralytic ileus, ascites	Heterozygotic prothrombin mutation	2	3	26	776	Yes
2	43/M	PT	Acute	Abdominal pain	None	7	17	56	663	Yes
3	25/M	PT	Acute	Abdominal pain	Heterozygotic factor V Leiden mutation	4	15	32	610	Yes
4	48/M	PT	Acute	abdominal pain	Splenectomy, hemophagocytic lymphohistiocytosis	14	18	57	594	No
5	74/M	PT	Acute	None	Inflammation	1	19	19	n.a	Yes
6	19/F	PT	Acute	Abdominal pain, diarrhea, vomiting	Oral contraceptives, exsiccosis	10	11	58	451	No
7	57/F	TIPS	Acute	Mild abdominal pain	Diabetes	42	49	17	399	Yes
8	26/M	TIPS	Acute	Variceal bleeding	Portal hypertension	1	5	27	922	Yes
9	57/F	TIPS	Acute	Abdominal pain, vomiting	JAK2 mutation	4	4	56	750	No
10	60/M	PT	Chronic	Portal hypertensive gastropathy, epigastric pain	Antiphospholipid syndrome	Elective	5	11	3	Yes
11	73/M	PT	Chronic	Therapy resistant ascites, spontaneous bacterial peritonitis, esophageal varices	JAK2 mutation	Elective	2	12	420	No
12	51/M	TIPS	Acute	Gastric varices	disseminated intravascular coagulation (DIC)	0	6	68	45	Yes
13	51/M	TIPS	Acute	Abdominal pain	None	3	2	11	330	Yes
14	78/M	TIPS	Chronic	Therapy resistant ascites, spontaneous bacterial peritonitis, esophageal varices	None	Elective	7	85	30	No
15	56/F	TIPS	Chronic	Esophageal varices, hematemesis	Medicinal	3	22	66	14	Yes
16	68/M	PT	Chronic	Abdominal pain, esophageal varices, hematemesis	Chronic pancreatitis	1	12	17	480	Yes
17	41/F	TIPS	Chronic	Portal hypertensive gastropathy, epigastric pain, nausea, vomiting	IgG4-Related Sclerosing Cholangitis	Elective	2	12	7	Yes
18	41/M	TIPS	Acute	Abdominal pain, nausea, Constipation	Homozygotic Factor V Leiden mutation	29	1	9	270	Yes
19	24/M	TIPS	Chronic	esophageal varices, hematemesis	JAK2 mutation	1	10	18	42	Yes
20	29/M	TIPS	Acute	Abdominal pain	Heterozygotic prothrombin mutation	0	16	33	55	Yes
21	41/M	TIPS	Acute	Abdominal pain	Vaccine related thrombosis	0	7	29	54	Yes

n.a., not applicable (patient died in the course of therapy); JAK2, janus kinase 2; PT, percutaneous access; TIPS, transjugular intrahepatic portosystemic shunt

### Outcomes

**Fig. 2:**
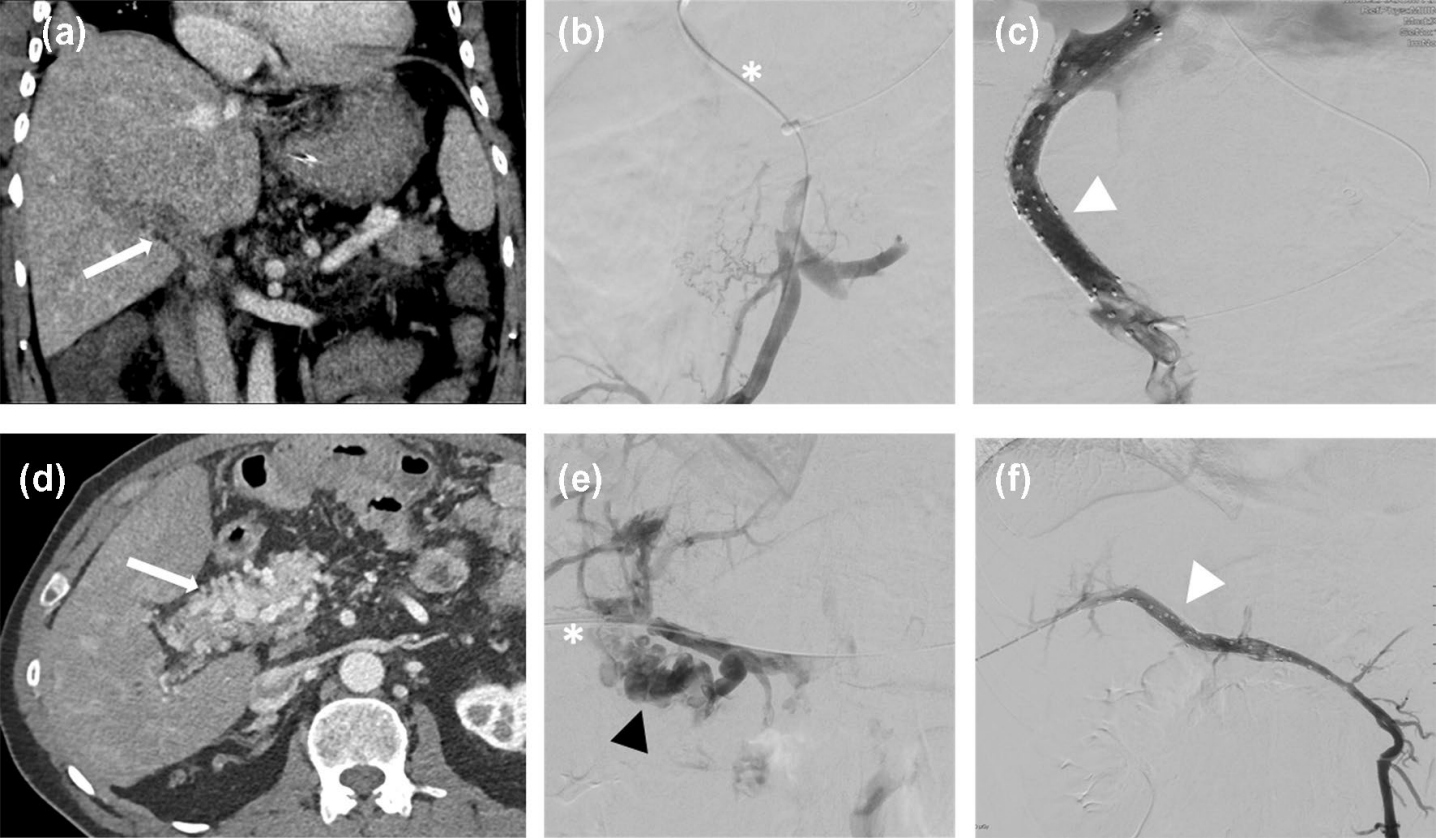
Exemplary cases of two patients with PVT included in this study who were treated with different recanalization techniques that include two interventional access routes (PT- and TIPS access). The first patient aged 50 (patient number 12/a–c) developed acute 2 PVT due to sepsis. **a** Initial CT with complete thrombotic obstruction of the extra- and intrahepatic portal venous system (white arrow). **b** Initial angiogram after TIPS access (asterisk) showing obstruction of the portal vein and the subsequent collateralization. **c** Angiogram showing successful patency of the portal vein achieved after thrombectomy, TIPS-Implantation and stenting up into the superior mesenteric vein (white arrowhead). The second patient aged 60 (patient number 10/d-f) with chronic portal vein thrombosis first diagnosed in 2011 and subsequent cavernous transformation (grade 3). Interventional recanalization was performed due to unsuccessful anticoagulation therapy and escalating portal hypertensive hemorrhagic gastropathy. **d** CT scan showing complete thrombotic obstruction of the portal vein with subsequent cavernous transformation. **e** Initial angiogram after percutaneous transhepatic access (asterisk) showing the cavernous transformation of the portal vein (black arrowhead). **f** Angiogram showing the successful patency of the portal vein achieved after porto-mesenteric stenting (white arrowhead). *CT* computed tomography, *PVT* portal vein thrombosis, *TIPS* transjugular intrahepatic portosystemic shunt

Median time from symptom onset to first intervention in patients with acute grade 2 PVT was 13 days (range of 3–49 days). Nine (42.8%) patients had initial recanalization via PT access, and twelve (57.2%) patients via TIPS access. Procedural details of each patient are given in Table 3. Three patients with initial PT access underwent sequential recanalization via transjugular access with secondary TIPS placement due to initially insufficient recanalization via the percutaneous access (defined as > 25% residual thrombosis of the portal vein or intrahepatic branches) (Table 4). The complete technical success rate was 55.5% in patients with initial PT access and 83.3% in patients with initial TIPS access (*p* = 0.331). However, the creation of a TIPS was significantly associated with higher technical success (86.6% vs. 33.3%, *p* = 0.030). None of the other procedure-related parameters correlated with technical success (Table 5). One patient in the percutaneous group had partial recanalization only. Due to poorer flow restoration via the percutaneous access, higher amounts of thrombolytics [recombinant tissue-Plasminogen Activator (rt-PA)] were required to restore flow via percutaneous access as the first procedure (mean 77.3 mg vs. 25.6 mg, *p* = 0.037) (Fig. 2).

**Table 3:** Procedural characteristics

Patient number	Route of intervention	Lysis	Rheolytic thrombolysis	Visceral stenting	TIPS creation	Additional measures
1	PT	Yes	No	No	No	
2	PT	Yes	No	SMV, VS	Yes	
3	PT	Yes	Yes	VMS	No	
4	PT	Yes	Yes	No	Yes	
5	PT	Yes	No	VS	No	
6	PT	Yes	Yes	No	No	
7	TIPS	Yes	Yes	VS	Yes	
8	TIPS	Yes	Yes	No	Yes	
9	TIPS	Yes	No	No	Yes	
10	PT	No	No	PV	No	
11	PT	No	No	No	No	
12	TIPS	No	No	SMV, VS	Yes	
13	TIPS	Yes	No	No	Yes	
14	TIPS	No	No	SV	Yes	
15	TIPS	No	No	No	Yes	
16	PT	No	No	SMV	Yes	
17	TIPS	No	No	No	Yes	
18	TIPS	Yes	No	SMV	Yes	
19	TIPS	No	No	No	Yes	
20	TIPS	Yes	No	Yes	Yes	Splenic vein puncture to guide TIPS creation
21	TIPS	Yes	No	No	Yes	

PT, percutaneous access; TIPS, transjugular intrahepatic portosystemic shunt; SMV, superior mesenteric vein; SV, splenic vein

**Table 4:** Comparison of procedural details

	PT access	TIPS access	*p*-value
Primary TIPS placement	0/9	12/12	
Secondary TIPS placement	3/9	0/12	
Stenting	5/9	5/12	0.669
PV stenting	2/9	2/12	
SMV stenting	3/9	3/12	
VS stenting	2/9	3/12	
Complete technical success	5/9	10/12	0.331
Thrombolysis	6/9	5/12	0.386
Mean rt-PA-lysis (mg)	77,3	25.6	0.037*

*PT* percutaneous, *TIPS* transjugular intrahepatic portosystemic shunt, *PV* portal vein, *SMV* superior mesenteric vein, *VS* splenic vein, *rt-PA* recombinant tissue-Plasminogen Activator

**Table 5:** Factors associated with technical success

*n* = 21	Yes (*n* = 15)	No (*n* = 6)	*p*-value
Gender (male)	11/15	4/6	> 0.99
Myeloproliferative etiology (yes)	6/15	3/6	> 0.99
Acute PVT	12/15	4/6	0.597
First approach (Percutaneous)	5/15	4/6	0.331
Extension into intrahepatic portal branches	10/15	4/6	> 0.99
Involvement of SMV	11/15	6/6	0.280
Involvement of SV	8/15	4/6	0.659
Thrombolysis (yes)	8/15	4/6	0.659
TIPS (yes)	13/15	2/6	0.030*
Rheolytic thrombolysis (yes)	3/15	2/6	0.597
Visceral stenting (yes)	8/15	3/6	> 0.99

Nine (42.8%) patients experienced hemorrhagic complications. Of these, five patients had bleeding from the liver, one from spleen due to additional splenic access, one from the stump of the splenic artery, and the other one had disseminated abdominal bleeding without localized arterial extravasation. In univariate analysis, thrombolysis was significantly associated with bleeding (88.8% vs. 33.3%, *p* = 0.024). Also, the PT group had higher bleeding than the TIPS group (66.6% vs. 25%, *p* = 0.087). Multivariate analysis showed both thrombolysis (*p* = 0.049) and PT access (*p* = 0.045) were independent significant risk factors of bleeding (Table 6). Three patients in the PT group and one patient in TIPS group underwent further surgery with packing and evacuation of hematoma (*p* = 0.272). No significant difference was observed in technical success (*p* = 0.597) or bleeding complications (*p* = 0.338) between acute grade 2 and chronic grade 3 PVT with presence of cavernous transformation (Table 5).

**Table 6:** Risk factors for bleeding

*n* = 21	Bleeding	No bleeding	*p*-value	Multivariate analysis
Gender (male)	6/9	9/12	> 0.99	–
Myeloproliferative etiology (yes)	5/9	4/12	0.396	–
Acute PVT	8/9	8/12	0.338	–
First approach (Percutaneous)	6/9	3/12	0.087	0.045*
Extension into intrahepatic portal branches	7/9	7/12	0.642	
Involvement of SMV	9/9	8/12	0.103	
Involvement of SV	7/9	5/12	0.184	
Thrombolysis (yes)	8/9	4/12	0.024	0.049*
TIPS (yes)	5/9	10/12	0.331	–
Rheolytic thrombolysis (yes)	2/9	3/12	> 0.99	–
Visceral stenting (yes)	3/9	7/12	0.387	–

*PT* percutaneous, *TIPS* transjugular intrahepatic portosystemic shunt, *PV* portal vein, *SMV* superior mesenteric vein, *VS* splenic vein, *rt-PA* recombinant alteplase

One patient from Center 1 with failed recanalization had bowel perforation and underwent resection of 20 cm of ileum. Three patients (14.2%) died during hospitalization. One patient died due to multiorgan failure and sepsis despite complete recanalization of PVT. One patient was lost due to intracerebral hemorrhage and hemorrhagic shock after hepatic bleeding. The other patient died after failed recanalization attempts due to sepsis.

### Patency

Primary patency (complete or partial) of the portal vein was reached in 16 patients (76.2%). The median follow-up was 365 days (range of 3–922 days). Two patients showed recurrence of PVT approximately after 30 weeks (one from each group). The patient from the TIPS group with recurrent PVT displayed minor thrombotic deposits after TIPS and stenting on invasive angiography and was treated with balloon angioplasty and local thrombolysis without any additional complications. All patients with a failed recanalization developed a chronic PVT with a subsequent cavernous transformation. None of the treated patients suffered from major long-term complications during the follow-up period; only one patient revealed mild gastroesophageal varices (grade I) on endoscopy. Follow-up laboratory values, clinical assessment, and ultrasound revealed no signs of cirrhosis formation.

## Discussion

Non-malignant, non-cirrhotic portal vein thrombosis is a rare but potentially fatal condition requiring rapid and efficient treatment. Although systemic anticoagulation is recommended throughout different therapy guidelines as the method of choice in treating portal vein thrombosis [8, 19], complete recanalization is only achieved in around 35% of the cases [20, 21]. Recent case series have shown that interventional therapies, such as thrombectomy, thrombolysis or TIPS, are effective in patients with acute [22, 23] or chronic PVT [24–29]. A systematic review of studies using TIPS in portal vein thrombosis showed a high rate of recanalization and long-term patency [30]. A recent multicentric study from Rössle et al. [13] comparing medical and interventional treatment in 65 patients showed low procedure-related mortality (2.9%) and a high success rate of the interventional treatment (17% vs. 54%). However, several techniques have been utilized in these studies, and the optimal interventional method needs to be defined. Our results have shown that the creation of TIPS resulted in significantly higher technical success, and percutaneous approach and thrombolysis are significantly associated with increased bleeding.

Non-malignant, non-cirrhotic portal vein thrombosis constitute a special entity, because in patients with underlying cirrhosis or malignancy, portal vein thrombosis usually develops in the long term, which allows more time to develop collaterals. Additionally, the treatment of the underlying disease is the mainstay in management of these patients, and thrombosis-directed therapy is mostly restricted to systemic heparin. It is also important to view cirrhosis of the liver as an independent risk factor for thrombosis, mainly because loss of liver function impacts both procoagulant and anticoagulant factors; therefore, a differential approach of therapy in the presence of cirrhosis is required [8].

To avoid acute and long-term complications, patients who do not benefit adequately from initial anticoagulation need to be offered more aggressive therapy options. In this study we describe an escalating invasive therapy regime, starting with thrombaspiration, followed by local thrombolysis (bolus and overnight lysis), rheolysis, balloon angioplasty, stenting, and possibly TIPS implantation, if the latter methods did not lead to adequate flow restoration. In the treatment of non-cirrhotic, non-malignant PVT, multiple studies with small cohorts have shown higher success rates for combined surgical/interventional and isolated interventional therapy than isolated anticoagulation therapy with recanalization rates ranging from 75% to 80%, respectively [11, 14, 31]. However, invasive recanalization is associated with significantly higher rates of major hemorrhagic complications, especially in patients who underwent invasive recanalization through a percutaneous transhepatic access [14]. In our case series, we compared two different access routes (PT vs. TIPS access) for PVT recanalization. In the past percutaneous access was predominantly used especially in case of one branch of the portal vein still being patent, facilitating the percutaneous puncture and allowing some degree of flow, whereas in extensive thrombosis of the entire portal venous system, TIPS is needed to achieve adequate flow restoration in order to maintain patency.

These recanalization routes offer different advantages and disadvantages. While recanalization of the portal vein through a percutaneous access allows the preservation of the endothelial wall and blood flow through the native portal vein and intrahepatic branches, recanalization via TIPS access requires the creation of an artificial connection. In patients with occluded intrahepatic branches, PT recanalization is more challenging, especially due to limited flow. In contrary, TIPS technique allows access to the portomesenteric system despite extension of thrombus in the intrahepatic branches, as seen in many PVT cases. Additionally, TIPS allows an easy access for further interventional procedures of the portal vein.

In this study, interventional recanalization of the portal vein through a transjugular approach showed significantly lower rates of major hemorrhagic complications. In this group, a significantly lower amount of thrombolysis was required to restore flow, while other procedural details such as use of additional stenting or rheolytic therapy were not associated with outcome. Percutaneous access requiring perforation of the liver capsule together with the increased amount of thrombolytics led to increased rates of major hemorrhagic complications. Previous studies of patients with impaired coagulation needing liver biopsy also showed higher complication rates after percutaneous access compared to the transjugular route [32]. Furthermore, probably due to stable outflow after TIPS, patients with TIPS had significantly higher clinical success.

Limitations of this analysis include the retrospective character of the study and a certain selection and procedural bias between the two centers. The yet limited follow-up time may underrepresent long-term benefits of PVT recanalization. Heterogeneity of onset of PVT poses another limitation, since acute on chronic PVT offers different challenges in management, yet with the focus of this study on the route to restore blood flow, the inclusion of both groups seems feasible. Additionally, low enrollment rates mainly due to interventional therapy not being the first-line therapy method in patients with non-cirrhotic, non-malignant PVT contribute to smaller study populations. With current guidelines increasingly recommending escalating recanalization therapy in patients affected by acute or chronic PVT and impending serious complications that are not manageable conservatively [2], further research and a greater sample size may be desirable.

## Conclusion

Invasive recanalization of the portal vein in carefully selected patients with PVT and absence of cirrhosis and malignancy offers a good therapeutic option with high recanalization and patency rates, compared to the moderate recanalization rates achieved by anticoagulation alone. The aim of invasive therapy is to avoid severe complications, both acute such as venous congestion of the gut, as well as chronic such as complications arising from portal hypertension, especially variceal bleeding. Although acute and chronic PVT offer different technical challenges, no significant difference in recanalization rates or periinterventional complications between the two forms of PVT were observed. Present data suggest that bleeding complications result predominantly from the percutaneous access and increased amounts of thrombolytics applied to restore proper flow. With low periinterventional morbidity and mortality and simultaneous high technical success rates and patency, portal vein recanalization in the TIPS technique is favorable and should be considered as the interventional treatment of choice.

## References

[1] Rajani R, Björnsson E, Bergquist A, Danielsson Å, Gustavsson A, Grip O (2010). The epidemiology and clinical features of portal vein thrombosis: a multicentre study. Alimentary pharmacology & therapeutics..

[2] Northup PG, Garcia-Pagan JC, Garcia-Tsao G, Intagliata NM, Superina RA, Roberts LN (2021). Vascular liver disorders, portal vein thrombosis, and procedural bleeding in patients with liver disease: 2020 practice guidance by the American Association for the Study of Liver Diseases. Hepatology..

[3] Dickson BC (2004). Venous thrombosis: on the history of Virchow’s triad. Univ Toronto Med J..

[4] Kumar A, Sharma P, Arora A (2015). portal vein obstruction–epidemiology, pathogenesis, natural history, prognosis and treatment. Alimentary pharmacology & therapeutics..

[5] Zocco MA, Di Stasio E, De Cristofaro R, Novi M, Ainora ME, Ponziani F (2009). Thrombotic risk factors in patients with liver cirrhosis: correlation with MELD scoring system and portal vein thrombosis development. Journal of hepatology..

[6] Noronha Ferreira C, Seijo S, Plessier A, Silva-Junior G, Turon F, Rautou PE (2016). Natural history and management of esophagogastric varices in chronic noncirrhotic, nontumoral portal vein thrombosis. Hepatology..

[7] Chen H, He C, Lv Y, Fan J, Tang S, Niu J (2020). Long-term results of variceal bleeding management in 302 patients with chronic extrahepatic portal vein obstruction. Journal of gastroenterology and hepatology..

[8] Simonetto DA, Singal AK, Garcia-Tsao G, Caldwell SH, Ahn J, Kamath PS. ACG Clinical Guideline: disorders of the hepatic and mesenteric circulation. Official journal of the American College of Gastroenterology| ACG. 2020;115(1):18–40. 10.14309/ajg.0000000000000486 31895720

[9] Hollingshead M, Burke CT, Mauro MA, Weeks SM, Dixon RG, Jaques PF (2005). Transcatheter thrombolytic therapy for acute mesenteric and portal vein thrombosis. Journal of vascular and interventional radiology..

[10] Hall TC, Garcea G, Metcalfe M, Bilk D, Rajesh A, Dennison A. Impact of anticoagulation on outcomes in acute non-cirrhotic and non-malignant portal vein thrombosis: A retrospective observational study. Impact of anticoagulation in acute non-cirrhotic and non-malignant portal vein thrombosis: A retrospective observational study. Hepato-gastroenterology. 2013;60(122):311–7. 23858545

[11] Klinger C, Riecken B, Schmidt A, De Gottardi A, Meier B, Bosch J (2017). Transjugular local thrombolysis with/without TIPS in patients with acute non-cirrhotic, non-malignant portal vein thrombosis. Digestive and liver disease..

[12] Wang C-Y, Wei L-Q, Niu H-Z, Gao W-Q, Wang T, Chen S-J (2018). Agitation thrombolysis combined with catheter-directed thrombolysis for the treatment of non-cirrhotic acute portal vein thrombosis. World journal of gastroenterology..

[13] Rössle M, Bettinger D, Trebicka J, Klinger C, Praktiknjo M, Sturm L (2020). A prospective, multicentre study in acute non-cirrhotic, non-malignant portal vein thrombosis: comparison of medical and interventional treatment. Alimentary Pharmacology & Therapeutics..

[14] Gerwing M, Wilms C, Heinzow H, Sporns PB, Heindel W, Schmidt H (2019). Escalating interventional recanalization therapy in non-cirrhotic, non-malignant acute portal vein thrombosis. European journal of gastroenterology & hepatology..

[15] Parikh S, Shah R, Kapoor P (2010). Portal vein thrombosis. The American journal of medicine..

[16] Malkowski P, Pawlak J, Michalowicz B, Szczerban J, Wroblewski T, Leowska E (2003). Thrombolytic treatment of portal thrombosis. Hepato-gastroenterology..

[17] Seijo S, Plessier A (2014). Noncirrhotic nontumoral portal vein thrombosis. Clinical liver disease..

[18] Öcal O, Stecher S-S, Wildgruber M. Portal vein thrombosis associated with ChAdOx1 nCov-19 vaccination. The Lancet Gastroenterology & Hepatology. 2021. 10.1016/S2468-1253(21)00197-7 PMC8186953 34115963

[19] Liver EAFTSOT. EASL Clinical Practice Guidelines: Vascular diseases of the liver. Journal of hepatology. 2016;64(1):179–202. 10.1016/j.jhep.2015.07.040 26516032

[20] Hall T, Garcea G, Metcalfe M, Bilku D, Dennison A (2011). Management of acute non-cirrhotic and non-malignant portal vein thrombosis: a systematic review. World journal of surgery..

[21] Plessier A, Darwish-Murad S, Hernandez-Guerra M, Consigny Y, Fabris F, Trebicka J (2010). Acute portal vein thrombosis unrelated to cirrhosis: a prospective multicenter follow-up study. Hepatology..

[22] Ferro C, Rossi UG, Bovio G, Centanaro M (2007). Transjugular intrahepatic portosystemic shunt, mechanical aspiration thrombectomy, and direct thrombolysis in the treatment of acute portal and superior mesenteric vein thrombosis. Cardiovascular and interventional radiology..

[23] Liu F-Y, Wang M-Q, Duan F, Wang Z-J, Song P (2010). Interventional therapy for symptomatic-benign portal vein occlusion. Hepato-gastroenterology..

[24] Fanelli F, Angeloni S, Salvatori FM, Marzano C, Boatta E, Merli M (2011). Transjugular intrahepatic portosystemic shunt with expanded-polytetrafuoroethylene-covered stents in non-cirrhotic patients with portal cavernoma. Digestive and Liver Disease..

[25] Klinger C, Riecken B, Schmidt A, De Gottardi A, Meier B, Bosch J (2018). Transjugular portal vein recanalization with creation of intrahepatic portosystemic shunt (PVR-TIPS) in patients with chronic non-cirrhotic, non-malignant portal vein thrombosis. Zeitschrift für Gastroenterologie..

[26] Han G, Qi X, He C, Yin Z, Wang J, Xia J (2011). Transjugular intrahepatic portosystemic shunt for portal vein thrombosis with symptomatic portal hypertension in liver cirrhosis. Journal of hepatology..

[27] Senzolo M, Tibbals J, Cholongitas E, Triantos C, Burroughs A, Patch D (2006). Transjugular intrahepatic portosystemic shunt for portal vein thrombosis with and without cavernous transformation. Alimentary pharmacology & therapeutics..

[28] Parvinian A, Bui JT, Knuttinen MG, Minocha J, Gaba RC (2014). Transjugular intrahepatic portosystemic shunt for the treatment of medically refractory ascites. Diagnostic and Interventional Radiology..

[29] Bilbao JI, Elorz M, Vivas I, Martínez-Cuesta A, Bastarrika G, Benito A (2004). Transjugular intrahepatic portosystemic shunt (TIPS) in the treatment of venous symptomatic chronic portal thrombosis in non-cirrhotic patients. Cardiovascular and interventional radiology..

[30] Rodrigues SG, Sixt S, Abraldes JG, De Gottardi A, Klinger C, Bosch J (2019). Systematic review with meta-analysis: portal vein recanalisation and transjugular intrahepatic portosystemic shunt for portal vein thrombosis. Alimentary pharmacology & therapeutics..

[31] Loss M, Lang SA, Uller W, Wohlgemuth WA, Schlitt HJ (2014). Combined surgical and interventional therapy of acute portal vein thrombosis without cirrhosis: a new effective hybrid approach for recanalization of the portal venous system. Journal of the American College of Surgeons..

[32] Atar E, Ari ZB, Bachar GN, Amlinski Y, Neyman C, Knizhnik M (2010). A comparison of transjugular and plugged-percutaneous liver biopsy in patients with contraindications to ordinary percutaneous liver biopsy and an “in-house” protocol for selecting the procedure of choice. Cardiovascular and interventional radiology..

